# Waking up to wearables: the next era of blood pressure measurement for primary care?

**DOI:** 10.3399/BJGP.2026.0181

**Published:** 2026-06-25

**Authors:** Sinéad Teresa Jennifer McDonagh, Rosina Cross, Jay Conor Rothery, Christopher Elles Clark

**Affiliations:** 1 Exeter Collaboration for Academic Primary Care (APEx), Department of Health and Community Sciences, Faculty of Health and Life Sciences, University of Exeter Medical School, St Luke’s Campus, Exeter, UK; 2 Ageing and long-term conditions, Genetics, and Epidemiology group, Clinical and Biomedical Sciences, University of Exeter Medical School, St Luke’s Campus, Exeter, UK

## The unresolved hypertension problem

Hypertension is the most prevalent modifiable cardiovascular risk factor and leading cause of morbidity and mortality.^1^ The prevalence of undiagnosed hypertension in England has been rising for over a decade, so finding people with uncontrolled hypertension, and treating them to target blood pressure (BP), are key to improving outcomes. Since 2011, the National Institute for Health and Care Excellence (NICE) has recommended ambulatory BP monitoring (ABPM) for the diagnosis of hypertension.^2^ Primary care uptake of, and access to, ABPM have proved variable, declining since the COVID-19 pandemic, with greater use and NHS support for home BP monitoring instead.^3^

## The rise of wearable technology

Novel technologies, typically integrated into watches, wristbands, or rings, offer new opportunities for self-measurement of BP and diagnosing hypertension. Many health trackers report BP trends with disclaimers against diagnostic use. In September 2025, the US Food and Drug Administration approved the Apple Watch Hypertension Notification Feature.^4^ Meanwhile, devices such as the Hilo wristband (Aktiia) or the CART BP ring (SkyLabs) have gained *conformité européenne* (CE) marks as BP measurement devices. Using photoplethysmography (PPG) to estimate BP, they either notify users of possible hypertension (Apple Watch) or report and summarise multiple 24-hour BP readings (Hilo and CART BP) via smartphone apps.

*“Novel technologies, typically integrated into watches, wristbands, or rings, offer new opportunities for self-measurement of BP and diagnosing hypertension.”*

The NHS 10 Year Health Plan positions technology-enabled care at the centre of future healthcare delivery using real-time health data. It envisages wearable devices becoming routine tools for remote cardiovascular monitoring by 2028, and integrated within preventive pathways by 2035. To realise these ambitions, primary care must be at the forefront of interpreting and responding to wearable generated BP data. This can only happen if healthcare professionals, regulators, and health systems prepare for their integration now.

Further technological innovations may follow; wearable beat-to-beat measurement is becoming feasible, offering further potential monitoring and diagnostic capabilities. For example, postural hypotension (PH), a common comorbidity of hypertension or its treatment, is a risk factor for falls. Currently poorly tested for in primary care, PH is influenced by various factors and can be poorly reproducible.^5^ Continuous wearable measurement can detect postural BP changes during activities of daily living, offering new opportunities to diagnose PH.^6^

## What are the challenges?

*Workload* — Uptake of wearable technology will promote patient-initiated BP measurement over the status quo of healthcare professional-initiated BP measurement. While empowering patients and promoting their engagement with cardiovascular risk management, multiple readings may promote anxiety and disrupt consultation patterns in primary care. Patients may present with alerts, trends, or large volumes of BP data from wearable devices. For already stretched primary care systems, this may further increase workload. We need clear guidance on integrating and interpreting wearable BP data into primary care.

Interpretation of results may not be straightforward. The Apple Watch, for example, generates alerts for a probability of hypertension, not actual BP readings. Watch users may justifiably seek advice following an alert, which could be difficult to provide without the accompanying BP values. Since the Watch’s sensitivities and specificities are modest, some alerts will be false positives, demanding otherwise unnecessary follow-up BP measurements to confirm or refute a hypertension diagnosis. This would represent an additional workload for primary care, risking anxiety for users awaiting confirmatory BP results. For this reason, a validated wearable BP measuring device, yielding BP data directly, open to sharing and review with healthcare professionals, may be preferable.^7^ Clear policy guidance for healthcare professionals is needed. For example, this could include only accepting BP data from validated CE-marked devices, and only for eligible patients (excluding special groups such as pregnancy or atrial fibrillation, for whom such devices are not validated).

*Data volume* — Most wearables report large numbers of BP readings (typically 30–50 per 24 hours), documented with bespoke summaries. At present, automated integration into clinical systems is unavailable, requiring work to add results to the record. Who will be responsible for interpreting these data? Methodologies for automated summaries may not match NICE guideline recommendations for diagnosis or monitoring.^2^ If data integration becomes automated, should clinical systems alert healthcare professionals and/or patients to abnormal BP readings?

Effectively summarised data will limit healthcare professional workload but will need careful design. Systems should offer flexibility to tailor summaries to individual needs, for example, average daytime BP may be wanted to diagnose hypertension (although equivalence to ABPM is not proven),^8^ whereas an overview of diurnal variation or postural BP changes would usefully inform assessment of postural symptoms following initiation or intensification of medication.

*Governance* — Questions arise regarding ownership, clinical responsibility, and liability for data. Without clear frameworks, healthcare professionals may be uncertain about the role of wearable data in clinical decision making. At what stage does consumer/patient-generated data become part of the medical record, and how should disagreements of interpretation between patient and healthcare professional be managed?

*Inequalities* — Currently, wearable technologies are accessible to those who can afford them, with broadband access, and digital literacy.^9^ Early adopters are generally health conscious — following healthy preventive lifestyles and aware of their BP. However, hypertension prevalence is rising in younger adults — key users of such new technologies.^1,10^ Underserved or socioeconomically disadvantaged groups, whose hypertension burden is greater, are at risk of exclusion from these developments. Wearables may also perform differently according to skin tones, presenting a novel ethnic aspect to device accuracy and validation.^11^ Thus, a market-led roll-out of new technology, without evidence-based policies, could widen existing cardiovascular health inequalities. PPG sensors are already widely used for wearable heart rate measurement. Future software updates could, therefore, enable a range of existing health-tracking devices to measure BP. Such a roll-out would align well with Core20PLUS5 framework aspirations (an evidence-based NHS England approach to reducing healthcare inequalities and improve life expectancy) to extend hypertension case findings to underserved groups, mitigating the need for costly new devices, thus improving reach and equity of access to this technology.

*Regulatory approvals* — Cuffless BP measurement could become integrated into almost any PPG-equipped wearable device, but what of their medicolegal status? Few commercially available wearable health trackers or watches are approved (CE marked or equivalent) medical devices for diagnosis. Such regulatory requirements increase manufacturer and consumer costs. This not only disincentivises such mass integration, but also perpetuates limited access for disadvantaged populations.

## Call to action

Wearable technology is advancing rapidly. Wearable BP measurement is the top-priority research recommendation of the British & Irish Hypertension Society (BIHS) and NICE falls guidance.^12,13^ International work adapting International Organization for Standardization (ISO) validation standards to wearable BP devices is well advanced.^14^ Meeting such standards will be an important step towards professional acceptance, but other actions are needed. Future research must evaluate impacts of wearables on workload, clinical decision making, and health inequalities.

In November 2025, the BIHS and IQVIA led a national multidisciplinary summit ‘Realising the Potential of Wearable Technology to Prevent Cardiovascular Disease’. This concluded that coordination across validation, data integration, performance, and equity are all essential to successfully benefit from these new technologies. To minimise impacts on workload, the summit proposed a single national standard for data entry into the NHS primary care record. The National Institute for Health and Care Research (NIHR) has also identified regulatory and data governance frameworks, overcoming challenges in accessibility, and clinical validation as key for safe and effective uptake.^10^

The pace of consumer uptake of wearables will not wait for primary care to be ready. The issue now is whether integration of these innovative technologies can be equitable and informed by evidence, or driven by market forces alone. Our immediate challenge is ensuring that primary care can respond.

*“… a market-led roll-out of new technology, without evidence-based policies, could widen existing cardiovascular health inequalities.”*

Achieving this will require a coordinated approach from the NHS, NIHR, NICE, and specialist societies, to a) clarify roles for wearable BP measurement within diagnostic pathways, and b) establish minimum validation and reporting standards for wearable technologies. Primary care must also engage with their local and regional services to ensure that these new tools for diagnosing hypertension are acceptable and effective. GPs will need to understand and trust wearable devices, to realise their potential to improve the hypertension care of the nation.
